# 

**Published:** 1895-01-12

**Authors:** 


### CONTENTS.

**Conculcation or Inoculation?** - 251
**The Early Symptoms of General Paralysis** - 252
**Modern Medico-Psychology and Psychiatry.** By T. S. Clouston, M.D. States of Defective Inhibition and of Uncontrollable Impulse (*continued*) - 253
**The Panacea in English Medicine.** II.—Gold - 254
**Medical Progress and Hospital Clinics:**
Diphtheritic Paralysis in Children - 257
Progress in Medicine.—Intestinal Diseases - 258
Progress in Surgery.—Cranial Surgery - 259
Spinal Surgery - 261
New Drugs and Preparations.—Ferratin - 262
**Annotations.**—Bacteria and their Products—De Motu Cordis et Sanguinis—The Prison as a Reformatory - 255
**Notes and News** - 256
**The Institutional Workshop:**
The Financial Strength of our Voluntary Charities - 263
Practical Departments.—A Typhoid Fever Bath - 264
The Story of the Insane from Year to Year - 264
Around the Hospitals - 265
Construction Notes - 266
**Scraps and Gleanings** - 266
**The Book World of Medicine and Science** - 267
**The Student of Medicine** - 267

### EXTRA SUPPLEMENT.—THE NURSING MIRROR.

**News from the Nursing World.**—Royal Gifts—Beware of Spurious Co-operations and Homes—Who Pays?—Infirmary Nursing—Nurses at Oxford—Unacceptable because Unfamiliar—Grateful Patients—West Country Guardians—New Year's Greetings at Glasgow—Progress in Cork—Very Young Midwives—Mental Nurses in U.S.A.—The Children's Home—Princess Louise at Chelsea—The Legion of Honour—Short Items - cix
**Elementary Anatomy and Surgery for Nurses.** By W. McAdam Eccles, M.B., B.S., F.R.C.S. I.—Introductory. cxi
**Addenbrooke's Hospital, Cambridge** - cxi
**Dress and Uniforms.**—The Brompton Hospital - cxii
**Queen Victoria's Jubilee Institute** - cxiii
**Entertainments** - cxiii
**Notes from India** - cxiv
**Wants and Workers** - cxiv
**Training of Probationers at Lambeth Workhouse Infirmary** - cxiv
**Where to Go** - cxiv
**Notes and Queries** - cxiv

